# Improved Production Rates of Hydrogen Generation and Carbon Dioxide Reduction Using Gallium Nitride with Nickel Oxide Nanofilm Capping Layer as Photoelectrodes for Photoelectrochemical Reaction

**DOI:** 10.1021/acsomega.4c03729

**Published:** 2024-07-26

**Authors:** Ching-Ying Sheu, Shih-Sian Tu, Shoou-Jinn Chang

**Affiliations:** † National Tainan Girl’s Senior High School, Tainan 70101, Taiwan; ‡ Department of Photonics, National Cheng Kung University, Tainan 70101, Taiwan; § Institue of Microelectronics and Department of Electrical Engineering, National Cheng Kung University, Tainan 70101, Taiwan

## Abstract

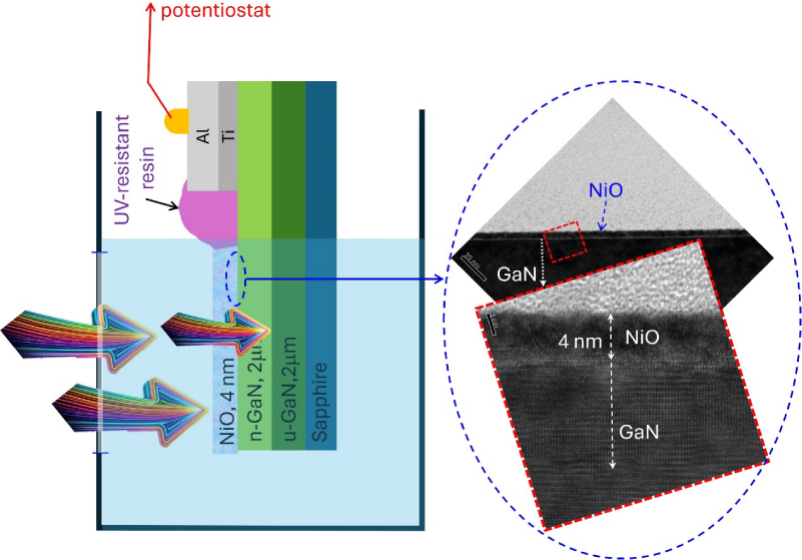

This work investigates the production of hydrogen (H_2_) and formic acid (HCOOH) through a photoelectrochemical (PEC) approach. Nickel oxide nanofilms prepared by sputtering capped on n-GaN photoelectrodes were employed to achieve simultaneous water photoelectrolysis and CO_2_ reduction. The study delves into the role of the nickel oxide layer, examining its potential as a catalyst and/or a protective layer. Furthermore, the influence of nickel oxide layer thickness on the performance of the photoelectrodes is explored. In essence, appropriate nickel oxide thickness is beneficial in increasing the photocurrent of the PEC reaction. The observed improvements in photocurrents and, hence, the production rates can be attributed to the functionality of the nickel oxide nanofilm: mitigating the negative influence of surface defects on n-GaN and facilitating the separation of photogenerated electron–hole pairs at the electrolyte/n-GaN interface. Specifically, PEC cells utilizing the 4 nm-thick nickel oxide nanofilms deposited on n-gallium nitride (n-GaN) electrodes demonstrate a significant enhancement in hydrogen and formic acid production rates. These rates were at least 45% higher compared to PECs using bare n-GaN electrodes.

## Introduction

Driven by rising greenhouse gas emissions, global warming has intensified in recent years, posing a growing threat to the long-term habitability of Earth for humans. In response, clean energy sources are recognized as a critical strategy to curb emissions. Among these, hydrogen energy holds immense potential due to its clean-burning nature. Current methods for hydrogen production include methane decomposition and water electrolysis.^1^ While the first method (referring to a previously mentioned process) generates greenhouse gas emissions, the second approach (referring to electrolysis) relies on renewable energy sources like solar or wind power to achieve a clean energy cycle, resulting in green hydrogen.^2^ Photoelectrochemical (PEC) hydrogen production shares similarities with electrolysis but utilizes solar energy to drive the reaction. However, this technology remains in its early stages and has not achieved commercial-scale production yet.^3^ On the other hand, the PEC reaction can not only split water into hydrogen and oxygen but also convert CO_2_ into useful products like CO, CH_4_, CH_3_OH, HCOOH, and other carbohydrates,^4,5^ etc. This solar-driven process essentially recycles greenhouse gases into valuable chemicals, making it a green technology. During solar-driven PEC reactions, semiconductors immersed in electrolytes act as photocatalysts. Light absorption by these semiconductors generates electron–hole pairs that drive water splitting. However, a major challenge is photocorrosion. This phenomenon occurs at the photoelectrode surface, leading to the formation of defects that promote the recombination of the generated electron–hole pairs, ultimately reducing photocurrent. Therefore, ideal semiconductors for high-performance and stable PEC applications require a specific set of properties.^6,7^

Gallium nitride (GaN)-based materials are promising candidates for photoelectrodes in solar-driven PEC hydrogen generation. However, several challenges hinder their practical application. Previous studies have shown that the rate of water oxidation is limited by the slow transfer of charge carriers at the interface between the electrolyte and n-GaN. This slow transfer results in the accumulation of holes at the n-GaN surface, leading to corrosion.^8^ While Waki et al. demonstrated water splitting with GaN under UV light irradiation,^9^ the material suffers from both instability in aqueous environments and the generation of excess current due to photocorrosion, limiting its long-term reliability.

Several strategies have been employed to enhance the rate of carrier exchange at the interface between the electrolyte and the n-type semiconductor. These strategies include the incorporation of a surface p–n junction^10^ or depositing a p-type metal oxide layer onto the photoanode.^11−13^ The surface p–n junction induces an upward band bending near the n-type semiconductor/electrolyte interface. This band bending effectively reduces the likelihood of electrons in the conduction band from entering the electrolyte while simultaneously increasing the driving force for holes to reach the electrolyte and participate in water oxidation at the photoanode.

Several research groups have investigated the application of NiO, a well-known p-type material, as a surface modifier for n-GaN photoanodes. This modification can serve a dual purpose: acting as a catalyst and offering protection against photocorrosion.^14−17^

The photocorrosion process at the GaN surface takes place via photogenerated electrons reaching the electrolyte/GaN interface. The reaction mechanism could be that the Ga atoms in GaN are reduced, and lattice nitrogens are released as NH_3_. The reaction equation could be assumed as follows.^18^
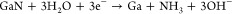


Another possibility is that the photogenerated holes travel to the GaN surface, leading to the formation of Ga_2_O_3_. The reactions can be described by the following equations.^19^
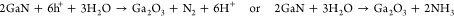


Then, the resulting Ga_2_O_3_ dissolves at the electrolyte/GaN interface, causing corrosion.

Considering the former case, NiO deposited on the GaN surface serves as an electron-blocking layer, preventing electrons from transferring to the GaN/electrolyte interface that reacted with the electrolyte. Thus, the reductive etching related to photogenerated electrons is suppressed. On the other hand, the photogenerated holes are swept to the NiO layer due to a type II heteroband edge and, thereby, diffuse to the electrolyte. However, most photogenerated holes will recombine with the defect states in the NiO layer during the diffusion process before they reach the electrolyte if the thickness of the NiO layer is larger than the diffusion length of the holes. Furthermore, NiO is highly resistive, and it is even a p-type material. If the p-NiO layer is too thick, it will likely prevent the GaN from working effectively as a photoanode. Therefore, the thickness of the NiO layer needs to be carefully controlled. Kamimura et al. observed a positive shift in the onset potential of NiO-modified GaN photoanodes with increasing NiO layer thickness grown by plasma-assisted molecular beam epitaxy.^18^ This suggests that thicker NiO layers provide enhanced surface protection for n-GaN anodes. This observation can be attributed to the inherent high resistivity of stoichiometric NiO films. The presence of nickel cation vacancies or interstitial oxygen within the NiO film creates a nonstoichiometric nickel oxide (NiOx). This NiOx can be further oxidized to form NiOOH, a material with significantly lower resistivity compared to NiO or nickel hydroxide (Ni(OH)_2_).^16^

The electrical properties of NiO thin films are also highly dependent on the fabrication method. Common techniques include the oxidation of metallic Ni films and sol–gel spin coating with NiO nanoparticles. However, these approaches often yield films with nonuniform thickness and compromised material quality. In general, n-GaN remains partially exposed to the electrolyte solution after NiO film deposition due to the use of either dispersed nanoparticles or porous films that are permeable to the electrolyte. Consequently, such NiO films offer inadequate protection for the underlying n-GaN layer. This study deposited dense NiO films onto the surface of n-GaN epitaxial films using a radio frequency sputtering technique with precise thickness control.

We fabricated composite thin films consisting of nickel oxide (NiO) deposited on n-GaN epitaxial films. These NiO/n-GaN composite films were then employed as photoelectrodes for PEC reactions. NiO films with varying thicknesses were deposited on both sapphire and n-GaN substrates to optimize the photoelectrodes. The electrical resistivity and light transmittance of these films were subsequently evaluated. The PEC experiments were carried out using an aqueous sodium chloride (NaCl) solution containing carbon dioxide (CO_2_) as the electrolyte. This configuration allowed for the simultaneous production of hydrogen and formic acid during light irradiation.^20^ Our results revealed that a thin NiO layer (approximately 4 nm) deposited on the n-GaN films significantly enhanced photocurrents while minimizing corrosion compared to pristine n-GaN photoelectrodes.

## Experimental Section

**Figure 1:**
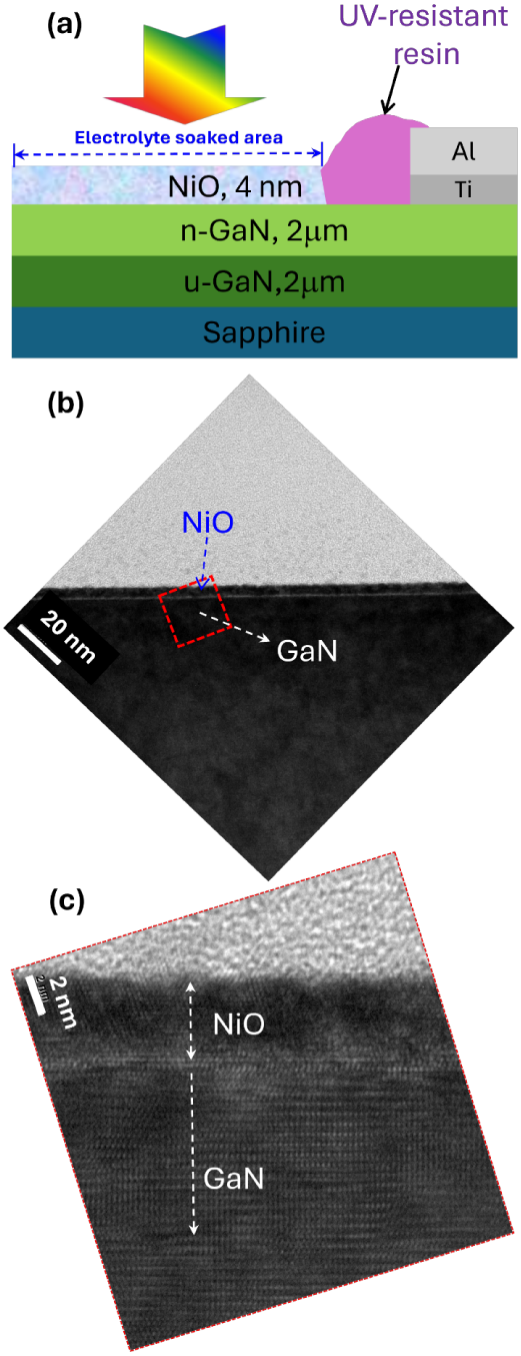
(a) Schematic structure of photoelectrodes made of NiO/n-GaN composite films. (b) A typical cross-section TEM image of the NiO/n-GaN composite films. (c) High-resolution TEM image taken from (b).

This study employed GaN epitaxial films grown on sapphire substrates using metal–organic chemical vapor deposition (MOCVD). The epitaxial structure consisted of a thin GaN nucleation layer (30 nm) deposited at 550 °C, followed by a thicker, undoped GaN layer (2 μm). A subsequently grown Si-doped GaN layer (n-GaN, 2 μm) was deposited on top at a higher temperature (1050 °C). The electron concentration in the n-GaN layer was measured to be 1 × 10^19^ cm^–3^. For the deposition of a nickel oxide (NiO) layer onto the n-GaN films, a mixture of argon and oxygen gases with flow rates of 90 and 10 standard cubic centimeters per minute (s.c.c.m), respectively, was introduced into the reactor during the sputtering process. The NiO film was grown on the n-GaN surface using an 80W radio frequency (RF) power applied to the NiO target. Subsequently, a wet etching process was employed to partially remove the NiO layer, thereby exposing a portion of the n-GaN surface for the subsequent deposition of a layered metal contact consisting of 50 nm of titanium (Ti) and 150 nm of aluminum (Al). Figure 1a depicts a schematic representation of the n-GaN photoelectrodes featuring a NiO capping layer. Figure 1b shows a representative transmission electron microscopy (TEM) image of a cross-section through the n-GaN film capped with a NiO nanofilm. The image reveals a dense and continuous NiO layer uniformly deposited on the n-GaN surface. Notably, the absence of any surface irregularities like pits or holes suggests a highly conformal deposition process. Figure 1c presents a high-resolution TEM image obtained from the region shown in Figure 1b. As shown in Figure 1c, the magnified lattice image reveals a regular lattice arrangement in some areas of the NiO layer, indicating a polycrystalline-like character of the sputtered NiO films on the n-GaN. This result is consistent with its high-resistivity property.^21−24^ Notably, even when the thickness of NiO films was increased under identical sputtering conditions, their resistivity remained remarkably high. In fact, it surpassed the maximum measurable limit of our instruments. To isolate the effect of the sputtered NiO cap layer on PEC performance, controlled n-GaN photoelectrodes were fabricated without the cap layer for comparison. A UV-resistant resin layer was incorporated between the Ti/Al electrical contact and the NiO cap layer (if present) or n-GaN working area to prevent the electrolyte from reaching metal contact. This exposed a working area of 1 × 1 cm^2^. During PEC experiments, an Xe lamp served as the light source, illuminating the n-GaN photoelectrodes with an irradiance density of 0.8 W/cm^2^. A potentiostat applied an external bias and measured the resulting photocurrents. A 1 M NaCl aqueous solution (pH 6.6) served as the electrolyte on the anode (photoelectrode) side. A separate compartment containing a Sn counter electrode (C.E.) and an Ag/AgCl reference electrode was immersed in 1 M NaCl solution under continuous CO_2_ bubbling in the cathode side. A cation exchange membrane (Nafion 117, Dupont) separated the C.E. compartment from the photoelectrode compartment to minimize the back reaction of dissolved species. Gas products from the PEC reactions were analyzed using a gas chromatograph (Agilent 7820A G.C.). The formic acid (HCOOH) production rate from CO_2_ reduction was verified and quantified using a liquid chromatograph (Agilent 1260 Infinity L.C.). A schematic diagram of the PEC reaction system is provided in Figure S1.

## Results and Discussion

**Figure 2:**
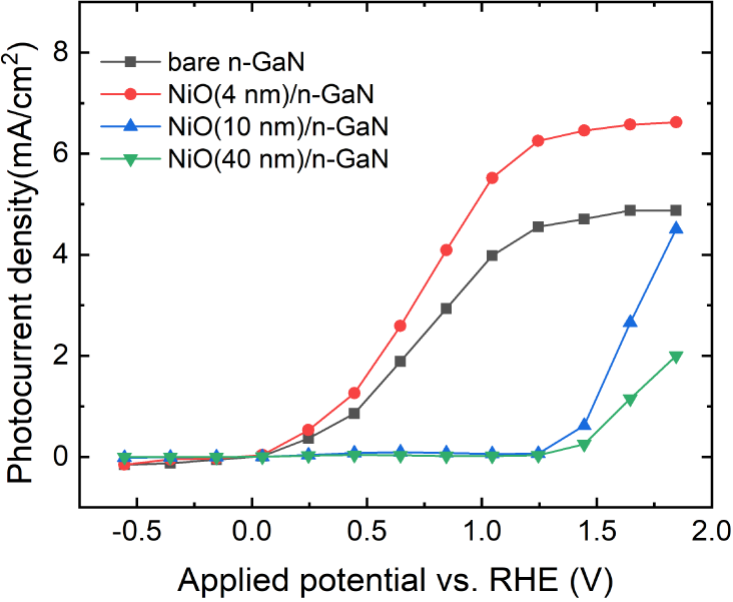
Typical LSV characteristics of different photoelectrodes as functions of applied potentials mean that the working electrode (WE) versus reference electrodes (RE).

Transition metal oxides (TMOs) are widely explored as cocatalysts in PEC applications. In general, the onset potentials observed in linear sweep voltammetry (LSV) curves decrease due to the surface loading of TMOs on the photoelectrodes. However, the inherent high resistivity of certain TMOs, like NiO, necessitates careful control of their thickness. Excessive thickness causes the increase of onset potentials. Prior studies by Kamimura et al.^18^ and our preliminary evaluations also suggested that thicker NiO capping layers negatively impacted the PEC performance of n-GaN photoelectrodes. Our research, as illustrated in Figure 2, demonstrates a decrease in typical photocurrents with increasing thickness of the NiO capping layer. This decrease can be explained by a reduced transfer efficiency of photogenerated holes due to the presence of the high-resistance NiO layer. Additionally, a thicker NiO cap layer may hinder light absorption by the underlying GaN material, even though NiO possesses a wide bandgap (Figure S2), reducing photocurrent. However, when the NiO layer was thinner, like 2 nm, the performance became almost indistinguishable from bare n-GaN photoelectrodes. Consequently, this article focuses on the PEC properties of n-GaN photoelectrodes capped with a 4 nm-thick NiO nanofilm (designated NiO/n-GaN) compared to bare n-GaN electrodes. As shown in Figure 2, the n-GaN/NiO photoelectrodes exhibit a similar onset potential to bare n-GaN. However, the saturation photocurrent is demonstrably higher. Although the present work employs a NiO layer with a thickness of approximately 4 nm to balance the protective function of NiO against surface defects of n-GaN and minimize its impact on charge transfer, it might still be insufficient to fully achieve the desired reduction in onset potential in the LSV curves. In other words, the sputtered NiO nanofilm does not significantly enhance the catalytic activity of photoelectrodes or reduce the energy barrier of the reaction.

Figure S3shows that the NiO/n-GaN photoelectrodes exhibit a more positive open-circuit voltage (OCV) under dark conditions than n-GaN photoelectrodes alone. This enhancement in OCV likely stems from the passivation of surface states on the n-GaN surface by NiO. This passivation reduces the pinning effect at the Fermi level, leading to a stronger band bending at the GaN surface. On the other hand, the creation of a type II heterojunction between the p-type NiO layer and the n-GaN to cause an increased band bending may be also a possible origin. In other words, the higher OCV reflects the upward band bending nature of the n-GaN photoanodes in dark equilibrium with the electrolyte. The passivation reduces the recombination of photogenerated carriers with surface defects, leading to an increase in photocurrent.

Furthermore, NiO possesses either a p-type character or significantly higher resistivity than n-GaN,^21−24^ leading to a larger energy band bending near the electrolyte/n-GaN interface, resulting in a stronger built-in electric field. This enhanced field effectively separates photogenerated carriers, ultimately increasing photocurrent.^10^ As shown in Figure S3, the NiO/n-GaN photoelectrodes exhibit a larger OCP of around 0.71 V, compared with bare n-GaN photoelectrodes (with an OCP of 0.49 V), which would lead to an enhanced electron–hole separation and hence a higher saturation photocurrent, as shown in Figure 2.

**Figure 3:**
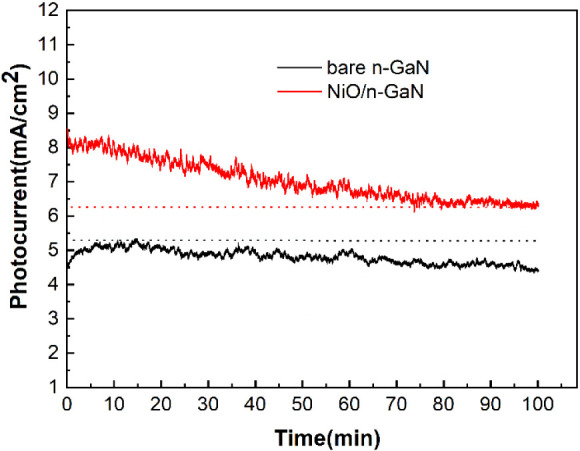
Photocurrent versus reaction time (*J*_ph_–*t*) for the studied photoelectrodes with and without the coating of NiO nanofilm. The *J*_ph_–*t* curves were conducted under a bias of 1 V (WE vs RE).

Our findings suggest that depositing a NiO nanofilm on the n-GaN surface significantly improves the PEC performance of the photoelectrodes. To assess the stability of these n-GaN-based photoelectrodes, we conducted a stability test in a three-electrode PEC system under a constant bias of 1 V. Details of the stability test parameters are provided in the experimental section. As shown in Figure 3, the typical photocurrent density of NiO/n-GaN photoelectrodes is higher than that of bare n-GaN electrodes. This is consistent with the photocurrent results obtained from the LSV scans presented in Figure 2. Although the photocurrents of NiO/n-GaN photoelectrodes show a significant decline in the early stage, they eventually level off. In contrast, the photocurrents of n-GaN photoelectrodes show a gradual decline in the late stage of the reaction time. This suggests that the photocurrents of n-GaN photoelectrodes will likely continue to decrease over time, whereas the photocurrents of NiO/n-GaN photoelectrodes may reach a plateau and remain relatively stable.

**Figure 4:**
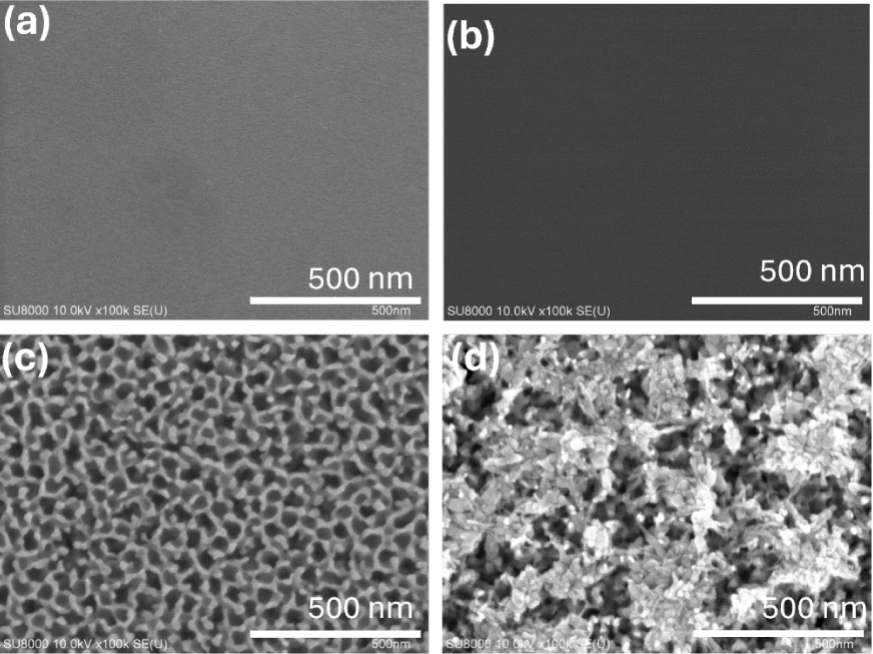
Typical FE-SEM images taken from the photoelectrodes made of bare n-GaN and NiO_2_/n-GaN. (a) and (c) display the surface morphologies of bare n-GaN photoelectrodes before and after the stability tests, respectively. (b) and (d) display the surface morphologies of NiO/n-GaN photoelectrodes before and after the stability tests, respectively.

To understand how the morphology of the photoelectrodes changed after the stability test, we employed scanning electron microscopy (SEM) to analyze their surface characteristics. Figure 4 shows representative SEM images of the photoelectrodes taken before and after the stability tests. Notably, the sputtered NiO nanofilm deposited on top of the n-GaN films displayed a featureless morphology, closely resembling the bare n-GaN, as shown in Figures 4a,b. This observation suggests the formation of a continuous and conformal NiO nanofilm on the n-GaN surface. This phenomenon contrasts with NiO layers prepared via other methods, such as oxidation of a metallic Ni nanofilm or synthesized through sol–gel method and precursor-based hydrothermal growth, typically resulting in isolated islands or a rough morphology characterized by densely packed nanoparticles.^25,26^ However, the surface of bare n-GaN after the stability tests was obviously corroded and showed dense holes at the surface, as shown in Figure 4c. Although some pits appear on the surface of NiO/GaN photoelectrodes, part of the NiO remains on the surface of NiO/GaN photoelectrodes, as shown in Figure 4d. This remaining NiO likely corresponds to regions with higher crystal quality, which aligns with the polycrystalline-like character of the sputtered NiO films observed in Figure 1c. In other words, the PEC reaction preferentially corrodes lower-quality areas within the NiO films, while the polycrystalline grains are more resistant to photocorrosion. This phenomenon may be the main reason for the difference in photocurrents during the stability tests; that is to say, part of the NiO covering the surface of n-GaN photoelectrodes continues to play the role of surface passivation or protection. On the other hand, during the PEC reaction process, the surface of n-GaN photoelectrodes has more structural defects due to the continuous emergence of corrosion pits, and these pits behaving as defect states will recombine with photogenerated carriers, thereby making the photocurrent of the bare n-GaN photoelectrodes continues to decline over the reaction time. In contrast, the photocurrents of NiO/n-GaN photoelectrodes will not likely change much in the late stage, as shown in Figure 3.

**Figure 5:**
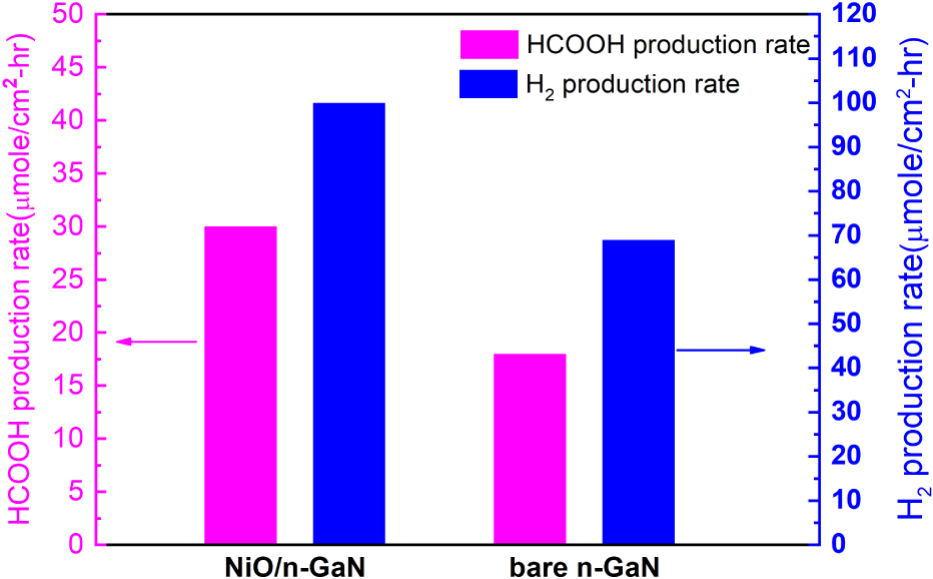
Production rates of H_2_ and HCOOH for the PEC reaction conducted at a bias of 1 V (WE vs RE). The total time for the PEC reaction was 100 min.

In addition to photocurrent measurements during the PEC reactions, the gaseous and liquid products collected within the PEC cells were also analyzed after the stability tests. While the existence of other carbohydrate products cannot be entirely ruled out in CO_2_-containing electrolytes, liquid chromatography (LC) analysis revealed that HCOOH was the primary product. Figure 5 shows the typical production rates of H_2_ and HCOOH for the PEC reaction conducted at a bias of 1 V. Notably, the typical production rates of H_2_ obtained from the PEC cells with photoelectrodes composed of NiO nanofilm on n-GaN exhibited a 45% enhancement compared to the bare n-GaN photoelectrodes. Similarly, the production rates of HCOOH for the NiO/GaN photoelectrodes demonstrated an approximate 66% increase. A comprehensive examination of the photoelectrodes has brought to light a notable incongruity. In other words, despite witnessing substantial increases in the production rates of HCOOH and H_2_ for the NiO/GaN photoelectrodes, the corresponding augmentation in photocurrents remained comparatively modest. Presently, a definitive rationale for this discrepancy remains elusive. However, speculative considerations posit that a portion of the current measured in the bare n-GaN photoelectrodes during the photoelectrochemical (PEC) reaction may originate from a contribution by corrosion current. This conjecture receives tentative support from the divergent cyclic voltammetry (CV) curves displayed in Figure S4. Significantly, the bare n-GaN photoelectrodes manifest a distinctive oxidation peak, while NiO/GaN photoelectrodes exclusively exhibit a reduction peak. Nonetheless, a conclusive elucidation of this phenomenon demands further experimental exploration. Table 1 benchmarks the PEC performance of our NiO/n-GaN photoelectrodes against other photoelectrodes made of NiO-coated semiconductors reported in the literature.

**Table 1:** A Benchmark of the PEC Performance of Our NiO/n-GaN Photoelectrodes against Other Photoelectrodes Made of NiO-Coated Semiconductors^a^

materials of photoelectrode	deposition method of NiO	electrolyte	light power density (mW/cm^2^)	photocurrent density (mA/cm^2^)	H_2_/HCOOH production rate	ref.
NiO/n-GaN	sol–gel drived	1 M NaCl	600 (Xe lamp)	∼0.9 at 1 V_(Ag/AgCl)_	15(H_2_) (μmol/h·cm^2^)	(16)
NiO/n-GaN	PA-MBE	1 M NaOH	100 (Xe lamp)	0.5–1.2 at 1.2 V_(RHE)_	N/A	(18)
NiO/n-GaN	diluted MOD	1 M NaOH	500 (Xe lamp)	2.6–3.1 at 0 V_(Ag/AgCl)_	N/A	(27)
NiO/n-GaN	spin-coated	1 M NaCl	700 (Xe lamp)	∼1.7 at 1.0 V_(Ag/AgCl)_	N/A	(28)
NiO/Si	thermal annealed Ni	0.5 M Na_2_SO_4_	100 (Xe lamp)	∼0.036 at 0 V_(Ag/AgCl)_	N/A	(29)
NiO/ZnO	electrodeposition	0.5 M Na_2_SO_4_	100 (Xe lamp)	1.8 at 1.23 V_(RHE)_	N/A	(30)
NiO/ZnO–CdS core–shell	chemical bath deposition	0.25 M Na_2_S and 0.35 M Na_2_SO_3_	100 (AM 1.5G)	1.8 at 1.23 V_(RHE)_	N/A	(31)
NiO/3C-SiC	pulsed-plasma sputtering	1 M NaOH	100 (AM 1.5G)	∼1.0 at 0.55 V_(RHE)_	27(H_2_) (μL/h·cm^2^)	(32)
NiO/n-GaN	RF sputtering	1 M NaCl	800 (Xe lamp)	∼7.0 at 1 V_(Ag/AgCl)_	30(H_2_)/100(HCOOH) (μmol/h·cm^2^)	this work

a N/A: not available.

## Conclusion

Based on the aforementioned experimental data and observations, our preliminary findings have demonstrated that n-GaN photoelectrodes covered with nickel oxide nanofilms deposited by sputtering exhibited enhanced photocurrent in photoelectrochemical (PEC) reactions. A similar trend was further corroborated by the analysis of the PEC reaction products (H_2_ and HCOOH). While nickel oxide nanofilms do not entirely avoid photocorrosion effects on n-GaN photoelectrodes, they demonstrate significant improvements compared to their bare counterparts. Future investigations could focus on optimizing the thickness and resistivity of the nickel oxide film to achieve superior protection for semiconductor photoelectrodes, particularly photoanodes.

## Supporting Information Available

Transmission of NiO on n-GaN, linear sweep voltammetry (LSV) curves, open-circuit voltages (OCV) versus time, cyclic voltammetry (CV) curves (PDF)

## References

[ref1] Hrycak B.; Mizeraczyk J.; Czylkowski D.; Dors M.; Budnarowska M.; Jasiński M. Hydrogen production by the steam reforming of synthetic biogas in atmospheric-pressure microwave (915 MHz) plasma. Sci. Rep. 2023, 13, 2204 10.1038/s41598-023-29433-y. 36750627 PMC9905514

[ref2] Stöckl F.; Schill W.-P.; Zerrahn A. Optimal supply chains and power sector benefits of green hydrogen. Sci. Rep. 2021, 11, 14191 10.1038/s41598-021-92511-6. 34244545 PMC8271012

[ref3] Patra K. K.; Bhuskute B. D.; Gopinath C. S. Possibly scalable solar hydrogen generation with quasi-artificial leaf approach. Sci. Rep. 2017, 7, 6515 10.1038/s41598-017-06849-x. 28747732 PMC5529526

[ref4] Atkins P.; Paula J. D. Elements of Physical chemistry, 6 th ed.; Oxford University Press, 2012.

[ref5] Xie S.; Zhang Q.; Liu G.; Wang Y. Photocatalytic and photoelectrocatalytic reduction of CO_2_ using heterogeneous catalysts with controlled nanostructures. Chem. Commun. 2016, 52, 35–59. 10.1039/C5CC07613G. 26540265

[ref6] Yu J. M.; Lee J.; Kim Y. S.; Song J.; Oh J.; Lee S. M.; Jeong M.; Kim Y.; Kwak J. H.; Cho S.; Yang C.; Jang J.-W. High-performance and stable photoelectrochemical water splitting cell with organic-photoactive-layer-based photoanode. Nat. Commun. 2020, 11, 5509 10.1038/s41467-020-19329-0. 33139804 PMC7606446

[ref7] Huang D.; Li L.; Wang K.; Li Y.; Feng K.; Jiang F. Wittichenite semiconductor of Cu3 BiS3 films for efficient hydrogen evolution from solar-driven photoelectrochemical water splitting. Nat. Commun. 2021, 12, 3795 10.1038/s41467-021-24060-5. 34145243 PMC8213846

[ref8] Sheu J.-K.; Liao P.-H.; Cheng H.-Y.; Lee M.-L. Photoelectrochemical hydrogen generation from water using undoped GaN with selective-area Si-implanted stripes as photoelectrodes. J. Mater. Chem. A 2017, 5, 22625–22630. 10.1039/C7TA07155H.

[ref9] Waki I.; Cohen D.; Lal R.; Mishra U.; Den Baars S. P.; Nakamura S. Direct water photoelectrolysis with patterned n-GaN. Appl. Phys. Lett. 2007, 91, 093519 10.1063/1.2769393.

[ref10] Fujii K.; Ono M.; Iwaki Y.; Sato K.; Ohkawa K.; Yao T. Photoelectrochemical Properties of the p–n Junction in and near the Surface Depletion Region of n-Type GaN. J. Phys. Chem. C 2010, 114 (51), 22727–22735. 10.1021/jp104403s.

[ref11] Chang X.; Wang T.; Zhang P.; Zhang J.; Li A.; Gong J. Enhanced Surface Reaction Kinetics and Charge Separation of p–n Heterojunction Co3O4/BiVO4 Photoanodes. J. Am. Chem. Soc. 2015, 137 (26), 8356–8359. 10.1021/jacs.5b04186. 26091246

[ref12] Zhong M.; Hisatomi T.; Kuang Y.; Zhao J.; Liu M.; Iwase A.; Jia Q.; Nishiyama H.; Minegishi T.; Nakabayashi M.; Shibata N.; Niishiro R.; Katayama C.; Shibano H.; Katayama M.; Kudo A.; Yamada T.; Domen K. Surface Modification of CoOx Loaded BiVO_4_ Photoanodes with Ultrathin p-Type NiO Layers for Improved Solar Water Oxidation. J. Am. Chem. Soc. 2015, 137 (15), 5053 10.1021/jacs.5b00256. 25802975

[ref13] Huang J.; Liu T.; Wang R.; Zhang M.; Wang L.; She H.; Wang Q. Facile loading of cobalt oxide on bismuth vanadate: Proved construction of p–n junction for efficient photoelectrochemical water oxidation. J. Colloid Interface Sci. 2020, 570, 89 10.1016/j.solmat.2020.110723. 32142906

[ref14] Gao F.; Tang X.; Yi H.; Zhang B.; Zhao S.; Wang J.; Gu T.; Wang Y. NiO-modified coconut shell based activated carbon pretreated with KOH for the high-efficiency adsorption of NO at ambient temperature. Ind. Eng. Chem. Res. 2018, 57, 16593–16603. 10.1021/acs.iecr.8b03209.

[ref15] Koike K.; Goto T.; Nakamura S.; Wada S.; Fujii K. Investigation of carrier transfer mechanism of NiO-loaded n-type GaN photoanodic reaction for water oxidation by comparison between band model and optical measurements. MRS Commun. 2018, 8, 480 10.1557/mrc.2018.51.

[ref16] Su C.-L.; Kogularasu K.; Yao Y.-T.; Liao P.-H.; Lee M.-L.; Sheu J.-K. Effect of KOH-Treatment at Sol–Gel Derived NiOx Film on GaN Photoanodes in Hydrogen Generation. ACS Appl. Energy Mater 2021, 4 (8), 8030 10.1021/acsaem.1c01345.

[ref17] Sun K.; Saadi F. H.; Lichterman M. F.; Hale W. G.; Wang H. P.; Zhou X.; Plymale N. T.; Omelchenko S. T.; He J. H.; Papadantonakis K. M.; Brunschwig B. S.; Lewis N. S. Stable solar-driven oxidation of water by semiconducting photoanodes protected by transparent catalytic nickel oxide films. Proc. Natl. Acad. Sci. U. S. A 2015, 112, 3612–3617. 10.1073/pnas.1423034112. 25762067 PMC4378389

[ref18] Kamimura J.; Budde M.; Bogdanoff P.; Tschammer C.; Abdi F. F.; van de Krol R.; Bierwagen O.; Riechert H.; Geelhaar L. Protection Mechanism against Photocorrosion of GaN Photoanodes Provided by NiO Thin Layers. Sol. RRL 2020, 4, 2000568 10.1002/solr.202000568.

[ref19] Murata J.; Okamoto T.; Sadakuni S.; Hattori A. N.; Yagi K.; Sano Y.; Arima K.; Yamauchi K. Atomically Smooth Gallium Nitride Surfaces Prepared by Chemical Etching with Platinum Catalyst in Water. J. Electrochem. Soc. 2012, 159, H417–H420. 10.1149/2.051204jes.

[ref20] Jiang M.; Wu H.; Li Z.; Ji D.; Li W.; Liu Y.; Yuan D.; Wang B.; Zhang Z. Highly Selective Photoelectrochemical Conversion of Carbon Dioxide to Formic Acid. ACS Sustainable Chem. Eng. 2018, 6, 82–87. 10.1021/acssuschemeng.7b03272.

[ref21] Poulain R.; Lumbeeck G.; Hunka J.; Proost J.; Savolainen H.; Idrissi H.; Schryvers D.; Gauquelin N.; Klein A. Electronic and Chemical Properties of Nickel Oxide Thin Films and the Intrinsic Defects Compensation Mechanism. ACS Appl. Electron. Mater 2022, 4, 2718 10.1021/acsaelm.2c00230.

[ref22] Liu Y.-H.; Liu X.-Y.; Sun H.; Dai B.; Zhang P.; Wang Y. Tuning the Electrical Properties of NiO Thin Films by Stoichiometry and Microstructure. Coatings 2021, 11, 697 10.3390/coatings11060697.

[ref23] Mohr J.; Hennen T.; Bedau D.; Nag J.; Waser R.; Wouters D. J. Fabrication of Highly Resistive NiO Thin Films for Nanoelectronic Applications. Adv. Phy. Res. 2022, 1 (1), 2200008 10.1002/apxr.202200008.

[ref24] Sato H.; Minami T.; Takata S.; Yamada T. Transparent conducting p-type NiO thin films prepared by magnetron sputtering. Thin Solid Films 1993, 236, 27 10.1016/0040-6090(93)90636-4.

[ref25] Ivanova T.; Harizanova A.; Shipochka M.; Vitanov P. Nickel Oxide Films Deposited by Sol-Gel Method: Effect of Annealing Temperature on Structural, Optical, and Electrical Properties. Materials 2022, 15 (5), 1742 10.3390/ma15051742. 35268971 PMC8910923

[ref26] Tran T. T. B.; Park E. J.; Son J. T. Optimization of Hydrothermal Synthesis of Nickel Oxide with Flower-Like Structure. Korean J. Chem. Eng. 2024, 41, 473 10.1007/s11814-024-00070-z.

[ref27] Kim S. H.; Ebaid M.; Lee J. K.; Ryu S.-W. Efficient photoelectrochemical water splitting by a doping-controlled GaN photoanode coated with NiO cocatalyst. Acta Mater. 2014, 79, 88–193. 10.1016/j.actamat.2014.07.032.

[ref28] Wang H.-K.; Kogularasu S.; Liao P.-H.; Yao Y.-T.; Lee M.-L.; Sheu J.-K. NiOx nanoparticles as active water splitting catalysts for the improved photostability of a n-GaN photoanode. Sol. Energy Mater. Sol. Cells 2020, 216, 110723 10.1016/j.solmat.2020.110723.

[ref29] Zhang F.-Q.; Hu Y.; Meng X.-M.; Peng K.-Q. Fabrication and photoelectrochemical properties of silicon/nickel oxide core/shell nanowire arrays. RSC Adv. 2015, 5, 88209–88213. 10.1039/C5RA13857D.

[ref30] Mao Y.; Cheng Y.; Wang J.; Yang H.; Li M.; Chen J.; Chao M.; Tong Y.; Liang E. Amorphous NiO electrocatalyst overcoated ZnO nanorod photoanodes for enhanced photoelectrochemical performance. New J. Chem. 2016, 40, 107–112. 10.1039/C5NJ01815C.

[ref31] Jian J.; Shi Y.; Ekeroth S.; Keraudy J.; Syvaj Arvi M. R. Y.; Sun U.; Helmersson J. A nanostructured NiO/cubic SiC p–n heterojunction photoanode for enhanced solar water splitting. J. Mater. Chem. A 2019, 7, 4721–4728. 10.1039/C9TA00020H.

[ref32] Iyengar P.; Das C.; Balasubramaniam K. R. Photoelectrochemical performance of NiO-coated ZnO–CdS core-shell photoanode. J. Phys. D: Appl. Phys. 2017, 50, 10LT01 10.1088/1361-6463/aa5875.

