# Supplementary material for: Improved Production Rates of Hydrogen Generation and Carbon Dioxide Reduction Using Gallium Nitride with Nickel Oxide Nanofilm Capping Layer as Photoelectrodes for Photoelectrochemical Reaction
Source: ACS Omega. 2024 Jul 26;9(31):33882–7. doi: 10.1021/acsomega.4c03729 (PMC11307980; doi:10.1021/acsomega.4c03729)
Supplement: Supplementary file 1 — ao4c03729_si_001.pdf [file ao4c03729_si_001.pdf]

Supporting information

Improved Production Rates of Hydrogen Generation and Carbon Dioxide Reduction using Gallium Nitride with Nickel Oxide Nanofilm Capping Layer as Photoelectrodes for Photoelectrochemical Reaction

Ching-Ying Sheu[†], Shih-Sian Tu [‡], and Shoou-Jinn Chang[‡,$,*]

[†]National Tainan Girl's Senior High School, Tainan, Taiwan
[‡]Department of Photonics, National Cheng Kung University, Tainan, Taiwan-70101.
[$]Institue of Microelectronics and Department of Electrical Engineering, National Cheng Kung University, Tainan, Taiwan
*E-mail: changsj@mail.ncku.edu.tw

Figure S1 displays a schematic PEC reactor using GaN-based photoelectrodes for hydrogen generation and CO_2 reduction in this study. The electronic grade NaCl (99.99 %) and deionized water (resistivity>15 M Ω·cm) were mixed to form 1 mole/L solution, which served as the electrolyte in the PEC reaction.

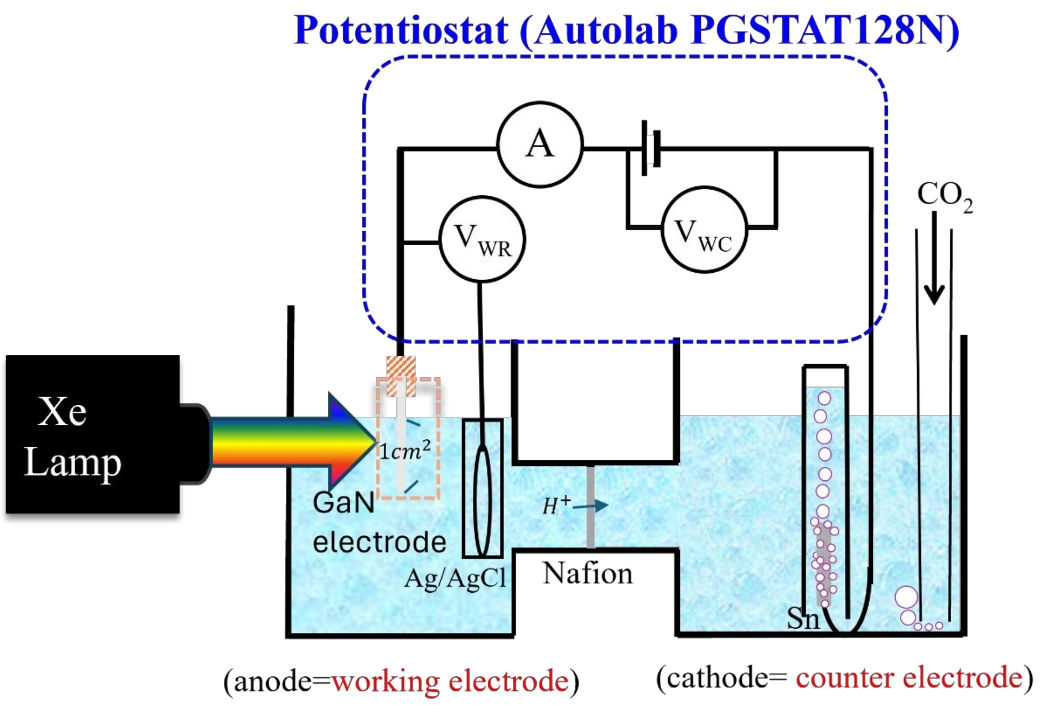

Fig. S1 A schematic PEC reaction system.

Figure S2(a) depicts representative transmission spectra for n-GaN samples with varying NiO overlayer thickness. The data reveals that for a 4 nm NiO nanofilm, the visible light transmittance remains nearly identical to bare n-GaN. However, a significant decrease in visible light transmittance is observed for n-GaN coated with a 40 nm NiO layer. The sharp drop in transmittance observed at 365 nm for all three samples in Figure S2(a) is primarily attributed to the bandgap absorption of n-GaN itself. To further isolate the influence of n-GaN on light absorption, the aforementioned NiO films were deposited on single-polished sapphires, and their transmission spectra were measured (Figure S2(b)). This approach eliminates the confounding factor of n-GaN absorption. The results confirm a substantially higher absorption for the 40 nm NiO film compared to the 4 nm nanofilm, particularly for incident photon energies exceeding the bandgap of n-GaN (wavelength < 365 nm).

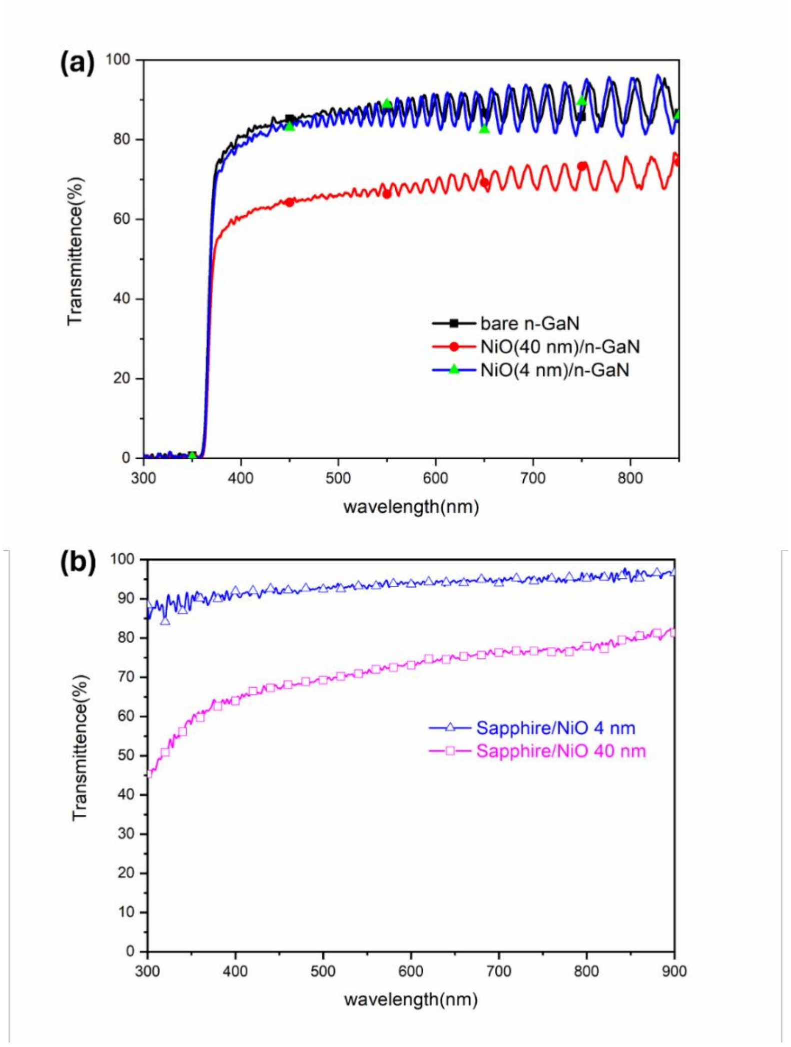

Fig. S2 (a)Representative transmittances taken from the n-GaN films, NiO/n-GaN composite films(b) Representative transmittances taken from the NiO thin films deposited on the substrate.

Fig. S3 depicts the representative open-circuit voltages (OCV) taken from the bare n-GaN and the NiO/n-GaN photoelectrodes.

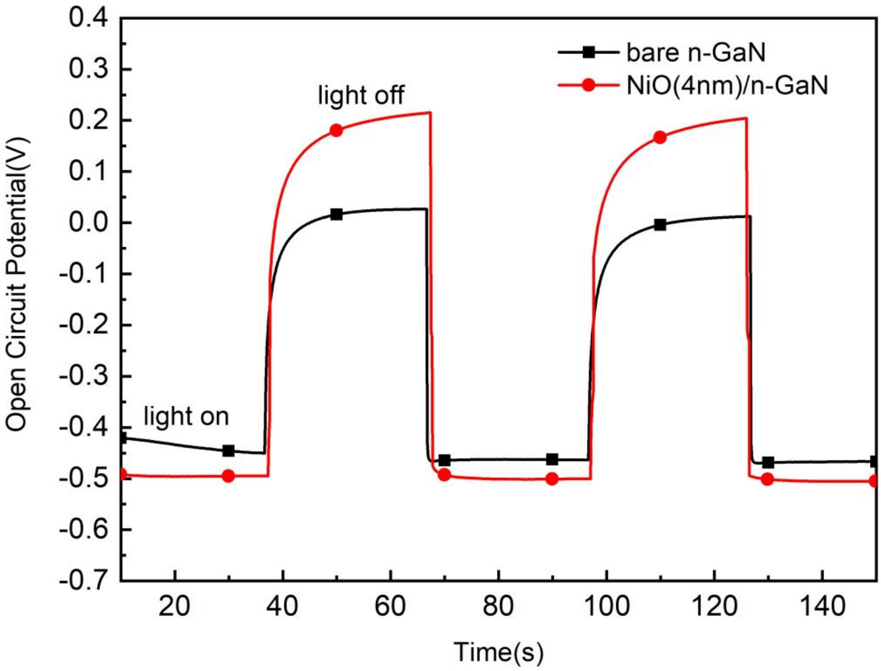

Fig. S3 Representative open-circuit voltages (OCV) taken from the bare n-GaN and the NiO/n-GaN photoelectrodes.

Cyclic voltammetry (CV) curves are presented in Figure S4. Notably, the bare n-GaN photoelectrodes exhibit both oxidation and reduction peaks. The presence of an oxidation peak suggests that the surface of the bare n-GaN undergoes corrosion during the photoelectrochemical (PEC) reaction. In contrast, the CV scans of the NiO/GaN photoelectrodes show only a reduction peak, and the oxidation peaks are absent. This observation implies that the NiO nanofilm deposited on the n-GaN surface passivates the surface defects, thereby reducing surface recombination and consequently mitigating the corrosion of the underlying n-GaN.

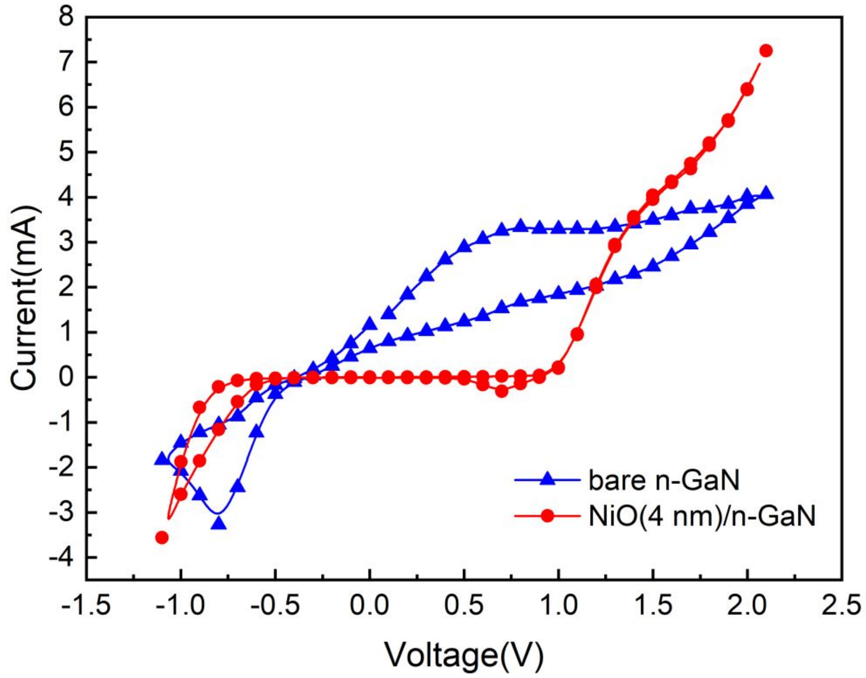

Fig. S4 Typical cyclic voltammetry (CV) curves taken from the bare n-GaN and NiO/GaN photoelectrodes.
